# Planar and single‐photon emission computed tomography imaging in dogs with thyroid tumors: 68 cases

**DOI:** 10.1111/jvim.15908

**Published:** 2020-09-26

**Authors:** Marit F. van den Berg, Sylvie Daminet, Emmelie Stock, Eva Vandermeulen, Stephanie Scheemaeker, Miguel Campos, Hans S. Kooistra, Sara Galac, Luc Duchateau, Kathelijne Peremans

**Affiliations:** ^1^ Department of Small Animals, Faculty of Veterinary Medicine Ghent University Merelbeke Belgium; ^2^ Department of Veterinary Medical Imaging and Small Animal Orthopaedics Ghent University Merelbeke Belgium; ^3^ Department of Clinical Veterinary Science, Vetsuisse Faculty Bern University Bern Switzerland; ^4^ Department of Clinical Sciences of Companion Animals, Faculty of Veterinary Medicine Utrecht University Utrecht The Netherlands; ^5^ Department of Nutrition, Genetics and Ethology, Faculty of Veterinary Medicine Ghent University Merelbeke Belgium

**Keywords:** canine, metastasis, scintigraphy, thyroid neoplasia

## Abstract

**Background:**

Information on scintigraphy findings in dogs with thyroid neoplasia is scarce. The use of single‐photon emission computed tomography (SPECT) could improve detection of metastatic disease.

**Hypothesis/Objectives:**

To describe planar and SPECT imaging findings in dogs with thyroid tumors, and to compare SPECT and thoracic radiography for metastasis detection.

**Animals:**

Sixty‐eight dogs with thyroid neoplasia.

**Methods:**

Retrospective study, search of medical records for dogs with thyroid neoplasia (2008‐2018).

**Results:**

Thyroid scintigraphy was available from 68 dogs, of which 6 presented after surgical resection. Radionuclide uptake was increased in 56% of dogs, decreased in 24%, and comparable to that of the salivary glands in 13%. The remainder had multiple masses with variable uptake. A homogeneous uptake pattern was present in 16% and a heterogeneous uptake pattern in 73%. In 11% (all dogs with multiple masses), various uptake patterns were present. Thyroid tumors were well delineated in 55%. There was a significant association between hormone status and uptake pattern (*P* = .009), with a heterogeneous uptake pattern in the majority of euthyroid dogs, and hormone status and tumor circumscription (*P* = .003), with well‐circumscribed margins in the majority of hypothyroid and hyperthyroid dogs. Thoracic SPECT imaging was available in 39 dogs and identified metastatic lesions in 15 dogs. Thoracic radiographs were performed in 14 of these dogs, and detected metastases in 3 dogs.

**Conclusions and Clinical Importance:**

SPECT imaging is a viable imaging technique to screen for thoracic metastasis and wider use of SPECT imaging is recommended in dogs with thyroid neoplasia.

**Abbreviations**
^123^I: iodine‐123
^131^I: iodine‐131
^99^mTcO_4_: pertechnetate
CT: computed tomography
FTC: follicular cell thyroid carcinoma
IHC: immunohistochemistry
MBq: megabecquerel
MTC: medullary thyroid carcinoma
SPECT: single‐photon emission computed tomography
T/S ratio: thyroid to salivary gland ratio
TSH: thyroid‐stimulating hormone
TT4: total thyroxine

## 1 INTRODUCTION

Thyroid tumors are among the most common endocrine tumors in dogs.^1^ The vast majority of thyroid tumors in dogs are malignant, and 16% to 60% of dogs have evidence of metastases at the time of diagnosis.^1^, ^2^, ^3^ Thyroid tumors can arise from follicular cells or from parafollicular C‐cells.^2^ Thyroid scintigraphy has an important added value in the diagnosis, staging, and treatment planning of thyroid carcinoma.^4^, ^5^, ^6^ It is used to assess if a patient is a good candidate for radioiodine treatment and can identify ectopic and metastatic thyroid tissue.^4^, ^6^, ^7^ Distant metastases can be detected with planar scintigraphy.^7^, ^8^, ^9^, ^10^, ^11^ In spite of this, planar scintigraphy is usually inferior to radiographs or computed tomography (CT) for the evaluation of pulmonary metastases.^5^, ^12^ Radioiodine (^123^I or ^131^I) might be superior to pertechnetate (^99m^TcO_4_) for detection of small or low intensity uptake lesions (eg, thoracic metastases) because of higher target‐to‐background ratios.^4^, ^5^ However, ^123^I is considerably more expensive than pertechnetate, which limits its use in veterinary medicine. Although ^131^I is less expensive and readily available, its relatively high radioactive burden to the patient, long half‐life (8.1 days) and poor imaging characteristics due to high energy γ‐rays not ideally suited to collimation by conventional gamma camera imaging, make it unfavorable for routine scanning.^4^, ^6^, ^13^ Both pertechnetate and radioiodine (^123^I and ^131^I) are actively concentrated or trapped by the thyroid gland, which is mediated by the sodium iodide symporter. However, unlike stable iodine and radioiodine, pertechnetate is not organified or incorporated into thyroid hormone by the thyroid gland, reflecting only the trapping mechanism, and not the “true” uptake of the thyroid gland.^13^, ^14^ After intravenous administration of radionuclides, some vascular activity inside the heart and blood vessels can be seen on scintigraphy, which is considered physiologic. Because this blood pool activity can be confused with pathologic radionuclide uptake, it can result in false‐positive results for metastatic disease.^15^

In humans with well‐differentiated thyroid carcinoma, planar ^131^I whole‐body scintigraphy is considered the routine diagnostic procedure and is used in the detection of both residual thyroid tissue and metastases for staging after thyroidectomy.^16^, ^17^, ^18^ Planar imaging, however, can give false‐negative results for small metastatic lesions. Single‐photon emission computed tomography (SPECT) has higher sensitivity and better contrast resolution than planar imaging, and can obtain cross‐sectional scintigraphic images, improving the detection of metastases.^4^, ^16^, ^17^, ^19^ The use of SPECT integrated with CT (SPECT/CT) systems provides an incremental value in staging and clinical management of humans with thyroid neoplasia because it provides more correct anatomic localization and characterization of abnormal foci of radioiodine uptake.^16^, ^17^, ^18^, ^20^ However, to the authors' knowledge, this technique has not been described in veterinary medicine.

The aim of this retrospective study was to describe planar and SPECT imaging findings in dogs with thyroid neoplasia, and to compare SPECT with thoracic radiography for detection of thoracic metastases.

## 2 MATERIALS AND METHODS

### 2.1 Case selection

Medical records of dogs referred to the Clinic of Small Animals of Ghent University for suspected or confirmed thyroid neoplasia between January 2008 and December 2018 were reviewed. All dogs had a confirmed diagnosis of thyroid neoplasia based on histopathology, cytology, abnormal radionuclide accumulation, or a combination of these. Dogs without scintigraphic examination were excluded.

### 2.2 Medical records review

Information obtained from the medical records included signalment, clinical signs, physical examination findings, serum total thyroxine (TT4) and thyroid‐stimulating hormone (TSH) concentrations, results of cytologic and histologic analyses and immunohistochemistry (IHC), imaging results (cervical scintigraphy, thoracic SPECT, thoracic radiographs, and CT), and treatment modality.

### 2.3 Scintigraphy

Dogs were injected with a median activity of 98 MBq (range, 41‐252 MBq) of ^99m^TcO_4_ (n = 53) or 37 MBq (range, 11‐48 MBq) of ^123^I (n = 17) via an intravenous catheter. Thyroid scans were acquired 20 to 30 minutes after injection with ^99m^TcO_4,_ and 24 hours after injection with ^123^I. A single static count based image (200 kcounts) was acquired with the dogs positioned in ventral recumbency, on a gamma camera equipped with a low energy high resolution collimator, located underneath the table (GCA 7200A; Toshiba, Tokyo, Japan). Matrix size was 256 × 256 and pixel size was 0.1 cm. Scan processing was performed using multimodality software (Hermes V5.0; Nuclear Diagnostics AB, Northfleet, UK). After the static acquisition, with the dogs remaining in ventral position, SPECT data of the thoracic area were acquired with a dual head gamma camera (GCA 7200A; Toshiba) equipped with low‐energy high resolution parallel hole collimators (tomographic resolution full width at half maximum = 9 mm). Data were acquired over a circular 360° rotation, for 20 minutes in step‐and‐shoot mode (120 steps, 10 seconds per step, 3° per step) on a 128 × 128 matrix. Images were then processed using iterative reconstruction and a Butterworth filter (cut‐off 1.4 cycli/cm, order 5). Pixel size was 1.72 mm.

In 8 dogs, scintigraphy was performed at a referring institution. For these dogs, planar scans were available for interpretation.

Thyroid scintigraphy was used to determine the location and radionuclide uptake of thyroid tumors. Compared to clinically healthy dogs, in which the appearance of the thyroid glands is characterized by homogeneous, symmetrical uptake by paired, oval thyroid lobes with smooth and regular margins in the midcervical area,^4^, ^5^, ^8^ the appearance of thyroid tumors was characterized by abnormal size and shape of the thyroid lobe(s), abnormal location of radionuclide uptake, and/or abnormal radionuclide uptake and uptake pattern.

The thyroid to salivary gland (T/S) ratio was calculated as the ratio of the mean counts per pixel of the thyroid region of interest to the mean counts per pixel of the ipsilateral parotid salivary gland region of interest. In case of a unilateral thyroid tumor, calculations were made for the affected thyroid lobe only, using the ipsilateral parotid salivary gland. In case of bilateral thyroid tumors, T/S ratios were determined separately for both thyroid lobes. For ectopic thyroid tumors, the T/S ratio was calculated using the average of the ratios compared to both parotid salivary glands.

Uptake patterns were described as (a) diffuse homogeneous radionuclide uptake throughout the thyroid tumor; and (b) irregular, heterogeneous uptake, which could be diffuse or could show (multi)focal areas of radionuclide accumulation with areas of increased and/or decreased uptake. The tumors were also characterized by their margins, with tumors having well‐defined or ill‐defined margins. Foci of uptake in the salivary glands and stomach were considered physiologic.

All images obtained by both planar acquisition and SPECT were analyzed by 1 board‐certified radiologist with extensive experience in nuclear medicine (K. Peremans) who was unaware of the clinical findings, of any other diagnostic imaging data, and of the histopathologic diagnosis.

### 2.4 Metastasis screening

Digital radiographic images (DDR, Eklin Medical Systems, Santa Clara, California) of the thorax, consisting of 2 or 3 views (right laterolateral, left laterolateral, and ventrodosal views), were reviewed for the presence of metastases. A high kilovoltage peak and low milliampere‐seconds technique was used, with exact values based on body size of the dog. For 4 dogs, thoracic radiographs were performed at a referring institution. For these dogs, digital radiographic images were available for interpretation.

Computed tomography images were obtained in sternal recumbency, in breath‐hold mode, using a 4‐slice helical CT device (Lightspeed Qx/I, General Electric Medical Systems, Chicago, Illinois). Contiguous slices were acquired at 2.5 mm thickness, 120 kV peak, 140 mA s, with an image matrix of 512 × 512. Computed tomography imaging was performed precontrast and after intravenous administration of nonionic ionated contrast medium Omnipaque (GE Healthcare BVBA, Diegem, Belgium). The images were reconstructed using conventional reconstruction algorithms (pulmonary and soft tissue algorithms). For evaluating the lung parenchyma, a lung window (width of 1400 hounsfield units [HU], level of −500 HU) was used, whereas a soft tissue window (width of 350 HU, level of 40 HU) was used for evaluation of mediastinal structures. Individual reviewers could adjust the window width and level of the images to personal preference.

Thoracic radiographs and CT images were reviewed by 2 radiologists (E. Stock and E. Vandermeulen), who were blinded to findings of related imaging tests, and agreement was reached by consensus.

SPECT images were reviewed for metastases by 1 board‐certified radiologist with extensive experience in nuclear medicine (K. Peremans) and results were compared to the medical records, of which the original reader was variable (both board‐certified radiologists and radiologists in residency program training). In case of disagreement with the medical records, a second board‐certified radiologist (E. Stock) reviewed the SPECT images, and agreement was reached by consensus.

### 2.5 Statistical analysis

Analyzed variables consisted of tumor scintigraphic uptake (increased, decreased, or comparable to the salivary gland based on visual evaluation of the scintigraphy images and T/S ratio), tumor scintigraphic uptake pattern (homogeneous or heterogeneous), tumor scintigraphic margins (well‐defined or ill‐defined), hormone status (euthyroid if serum TT4 was within reference range, hyperthyroid if serum TT4 was above reference range, and hypothyroid if serum TT4 was below reference range and serum TSH was increased), and tumor type (follicular cell thyroid carcinoma [FTC], medullary thyroid carcinoma [MTC], or unspecified thyroid [adeno]carcinoma). To determine if there were associations between variables (hormone status and tumor scintigraphic uptake, uptake pattern, tumor margins, and tumor type; tumor type and tumor scintigraphic uptake, uptake pattern, and tumor margins), the Fisher's exact test was applied at the 5% significance level using SAS Version 9.4 (SAS Institute, Inc, Cary, North Carolina).

## 3 RESULTS

### 3.1 Study population

A total of 105 dogs suspected or confirmed to have thyroid carcinoma were referred to our clinic during the study period. Of these, scintigraphy was performed in 75 dogs; however, 7 of 75 were excluded because a diagnosis of thyroid neoplasia could not be confirmed (n = 6) or scintigraphy images were not available (n = 1). The remaining 68 dogs were included in the study. Diagnosis of thyroid neoplasia was confirmed with histopathology (n = 38) and/or cytology (n = 30) in the majority of cases. In 8 dogs, the diagnosis of having a thyroid tumor was based on abnormal scintigraphy findings only.

Median age at the time of initial presentation was 9.8 years (range, 5.8‐17.8 years). Median body weight was 23 kg (range, 3.1‐67 kg). Of these 68 cases, 50% were female (25 neutered, 9 intact) and 50% were male (22 neutered, 12 intact).

Clinical signs at time of initial presentation included a palpable cervical mass (n = 63), weight loss (n = 18), cough (n = 14), polyuria/polydipsia (n = 13), respiratory signs (dyspnea, stridor; n = 9), dysphagia (n = 8), polyphagia (n = 6), reduced appetite (n = 6), exercise intolerance (n = 5), dysphonia (n = 4), weight gain (n = 2), regurgitation (n = 1), vomiting (n = 1), and diarrhea (n = 1).

Of 56 tested dogs, TT4 concentrations were within reference limits for 31 dogs and above reference range in 12 dogs. Nine dogs were hypothyroid at the time of initial presentation (based on serum TT4 concentrations below the reference range and increased serum TSH concentrations). Four dogs had low serum TT4 and normal serum TSH concentrations.

Of the 38 cases with histopathological reports (either after biopsy [core needle, surgical] or thyroidectomy), 25 were FTCs (follicular [n = 10], follicular‐compact [n = 9], compact [n = 6]), and 5 were MTCs. Of the remaining 8 tumors, 5 were reported as thyroid (adeno)carcinoma, 2 tumors could not be classified with certainty, and 1 adenoma was identified.

### 3.2 Thyroid scintigraphy

**FIGURE 1:**
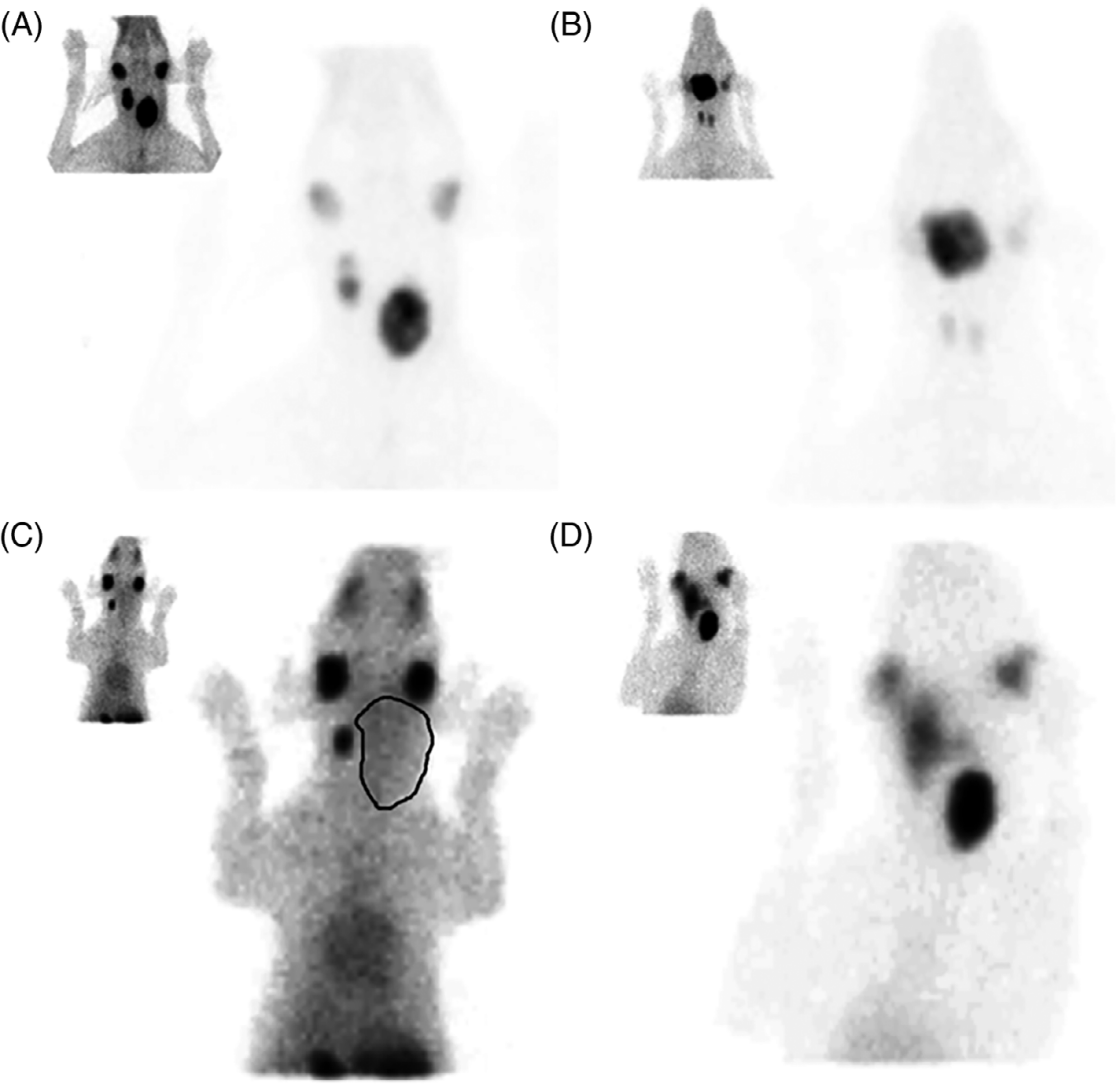
Ventral planar scintigraphic images of thyroid tumors in 4 dogs 20 to 30 minutes after intravenous injection with ^99m^TcO_4_, showing different uptake patterns, uptake extent, and tumor circumscriptions. A, Bilateral thyroid tumors with a heterogeneous uptake pattern, increased uptake, and regular tumor margins; B, Well‐circumscribed ectopic thyroid tumor with heterogeneous increased uptake; C, Unilateral (left) thyroid tumor with decreased homogeneous uptake and poorly circumscribed tumor margins. The tumor margin is manually outlined in the magnified image; D, Bilateral thyroid tumors with increased uptake. The left thyroid tumor has a homogeneous uptake pattern and is well delineated, whereas the right thyroid tumor has a heterogeneous uptake pattern and is poorly circumscribed

Thyroid scintigraphy was available from 68 dogs, of which 6 presented to our clinic after surgical resection. Data regarding the tumor location, tumor scintigraphic uptake, uptake pattern, and margins are presented in Table 1. Figure 1 reflects the different scintigraphic characteristics including degree of uptake, uptake pattern, and tumor circumscription.

**TABLE 1:** Tumor location, tumor scintigraphic uptake, uptake pattern, and margins for 68 dogs with thyroid tumors

Tumor location		Tumor scintigraphic uptake with T/S ratio			Tumor scintigraphic uptake pattern		Tumor scintigraphic margins	
Unilateral	39	Increased	3.2 (1.1‐12)	35	Homogeneous	10	Well‐defined	33
Bilateral	14	Decreased	0.73 (0.36‐0.90)	15	Heterogeneous	45	Ill‐defined	27
Ectopic	10	Comparable to salivary gland	0.96 (0.85‐1.0)	8	Multiple masses with variable uptake patterns	7	Multiple masses with variable margins	2
Unilateral + ectopic	1	Multiple masses with variable uptake		4				
Bilateral + ectopic	1							
Unclassified^a^	3							

*Note*: Values for T/S ratio are expressed as median and range.

Abbreviation: T/S ratio, thyroid to salivary gland ratio.

^a^ Tumor extent hindered accurate localization.

Of the 12 dogs with ectopic thyroid tumors, 8 had localized disease confined to the sublingual region, 1 had localized disease confined to the mediast, and 1 had abnormal radionuclide uptake at the level of the heart base. Two dogs had multicentric primary tumors; 1 had bilateral thyroid tumors in addition to sublingual ectopic disease, and 1 had a unilateral thyroid tumor in addition to sublingual ectopic disease.

There was no significant association between uptake extent and hormone status (*P* = .15) or tumor type (*P* = .27), although the majority of hyperthyroid dogs (10 of 12 dogs) had increased radionuclide uptake. Of the 5 dogs with MTCs, 2 showed decreased pertechnetate uptake, whereas uptake was increased in 2 dogs. In 1 dog, ^123^I scintigraphy was performed before and after administration of recombinant human TSH, which had increased tumor uptake after recombinant human TSH injection.

There was a significant association between uptake pattern and hormone status (*P* = .009), with a heterogeneous uptake pattern in 88% of the tumors of euthyroid dogs, 62% of tumors of hypothyroid dogs, and 46% of tumors of hyperthyroid dogs. No significant association was found between uptake pattern and tumor type (*P* = 1.0).

There was a significant association between tumor circumscription and hormone status (*P* = .003), but not tumor type (*P* = .42). The majority of tumors (85%) of hypothyroid and hyperthyroid dogs were well circumscribed, whereas the majority of tumors (60%) of euthyroid dogs had irregular margins. Of the dogs that showed thoracic metastases on SPECT imaging, half had irregular tumor margins.

### 3.3 SPECT imaging in comparison with thoracic radiographs and CT

**FIGURE 2:**
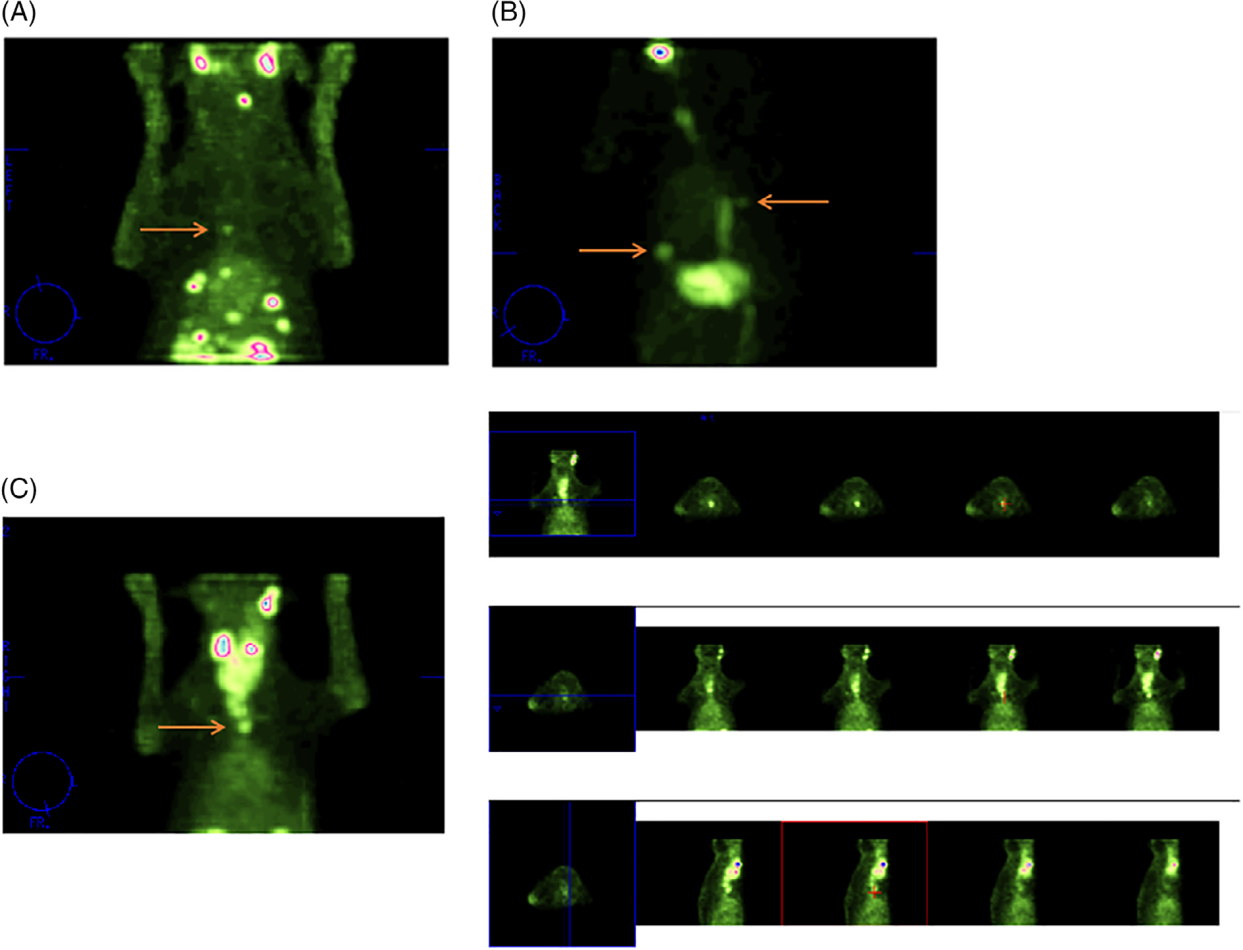
^99m^TcO_4_ SPECT imaging showing the presence of thoracic metastasis. A, Multiple foci in the caudal right and left lung field. One focus is located in the midline (arrow); B, Presence of 2 foci in the lung field (arrows), of which 1 is located dorsally to the heart at the midthoracic level and 1 in the left caudal lung field. Note the pertechnetate activity in the esophagus; C, Presence of foci in the midline of the cranial thorax. The images on the right are representing the different spatial oriented slices (transverse, dorsal, and sagittal slices). The cursor points to a focus of increased uptake in the thoracic midline

Results of SPECT imaging in comparison with thoracic radiographs and CT are presented in Table 2. Thoracic SPECT imaging was available in 39 dogs and identified thoracic metastatic lesions in 15 dogs (Figure 2). Pulmonary lesions were identified in 6 dogs, whereas lesions in the thoracic midline were present in 7 dogs. One dog had both thoracic midline and pulmonary lesions and 1 dog had a lesion at the level of the scapula based on SPECT imaging. The metastatic lesions that were identified by thoracic radiography (3 dogs) and CT (1 dog) in this group were all located in the lung field. The location of these metastatic lesions was consistent between SPECT and radiography, and between SPECT and CT. In 1 dog that had evidence of metastatic disease on SPECT imaging, both CT and radiographs were initially negative for thoracic metastases. However, radiologic follow‐up after 12 months revealed the presence of pulmonary metastases. In addition, repeat scintigraphy (both planar and SPECT imaging) showed progressive metastatic disease.

**TABLE 2:** Results of SPECT imaging in comparison with thoracic radiographs and CT for 39 dogs with thyroid tumors

SPECT		Thoracic radiographs				Thoracic CT			
		Number of dogs with radiographs performed	Metastasis	No metastasis	Doubtful	Number of dogs with CT performed	Metastasis	No metastasis	Doubtful
**Metastasis**	**15**	14	3	10	1	3	1	2	N/A
**No metastasis**	**20**	11	1	8	2	7	1	4	2
**Suspicious focus**	**2**	1	N/A	1	N/A	1	N/A	1	N/A
**Suspicion vascular activity**	**2**	2	N/A	1	1	1	1	N/A	N/A

Abbreviations: CT, computed tomography; N/A, not applicable; SPECT, single‐photon emission computed tomography.

In 20 dogs, SPECT did not show signs of intrathoracic metastasis. In 1 dog that did have radiographic evidence of metastatic disease, multiple pulmonary nodules were identified. In the 2 dogs with doubtful radiographic findings, CT was negative for metastatic disease. In 1 dog, CT showed metastatic disease (involvement of the cranial mediastinal and sternal lymph nodes, hepatic metastases, and 2 suspicious pulmonary nodules) that was neither identified on SPECT imaging nor thoracic radiographs. Two CTs were doubtful because they could not completely exclude the presence of metastatic disease due to the presence of atelectasis and motion artifact.

In 2 dogs, SPECT imaging showed foci of increased uptake in the thorax that were suggestive of vascular activity (normal blood pool activity), although metastatic disease could not be completely excluded. In 1 of these dogs, CT imaging was available and showed pulmonary metastases, whereas radiographs showed a suspicious lesion in the lung field. In 2 dogs, SPECT imaging showed a suspicious focus in the pulmonary field. In 1 of these dogs, radiographs showed no signs of metastatic disease. In the other dog, CT was performed, which did not detect thoracic metastases.

**FIGURE 3:**
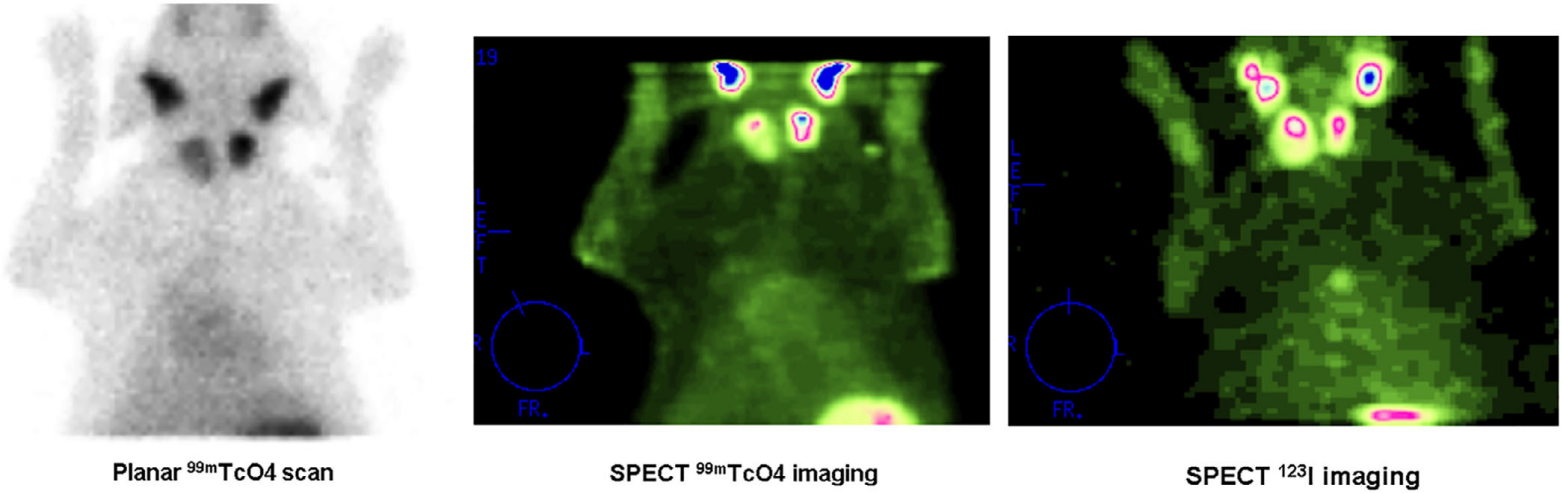
Planar (^99m^TcO_4_) and SPECT (^99m^TcO_4_ and ^123^I) imaging of a dog with bilateral thyroid tumors. ^123^I SPECT imaging revealed a thoracic metastasis that was not clearly identified with ^99m^TcO_4_ planar and SPECT imaging

In the majority of dogs, scintigraphy was performed using ^99m^TcO_4_. In 6 dogs, both ^99m^TcO_4_ and ^123^I planar scintigrams were performed, which showed no differences in tumor uptake or uptake pattern. In 2 of these dogs, thoracic SPECT was performed using both ^99m^TcO_4_ and ^123^I. In 1 dog, ^123^I SPECT imaging revealed a thoracic metastasis that was not clearly identified with ^99m^TcO_4_, whereas thoracic metastases were visible in both scans for the other dog, although better visualized on ^123^I SPECT imaging (Figure 3).

### 3.4 Follow‐up thyroid scintigraphy

Follow‐up scintigraphy was available in 14 dogs (all ^99m^TcO_4_ scans). Six dogs had follow‐up scintigraphy after thyroidectomy, which showed no uptake at the level of the surgery site except in 1 dog that showed remnant tissue after partial hyoidectomy of a sublingual ectopic tumor. In the remaining 8 dogs, follow‐up scintigraphy was available after initial radioiodine treatment. One dog showed no evidence of radionuclide uptake in the area of the tumor 6 weeks and 7 months after radioiodine treatment. A reduction in radionuclide uptake compared with pretreatment images was seen in 5 dogs. No change in radionuclide uptake was seen in 1 dog, and enlargement of the original tumor on follow‐up scintigraphy was noted in 1 dog.

## 4 DISCUSSION

Literature on planar ^99m^TcO_4_ and ^123^I scintigram findings in dogs with thyroid neoplasia is scarce, and nearly absent for SPECT imaging, which is only briefly mentioned as a thyroid imaging technique in 2 articles.^4^, ^19^ Our study describes planar and SPECT imaging in a large number of dogs with thyroid tumors and shows that SPECT imaging is a viable imaging technique to screen for thoracic metastasis. Wider use of SPECT is recommended complementary to structural imaging modalities such as CT and thoracic radiography.

Similar to previous studies,^8^, ^12^, ^14^, ^21^ the majority of dogs in our study had a heterogeneous uptake pattern. Two uptake patterns in dogs with thyroid tumors were previously described, differentiating a well‐circumscribed, homogeneous area of uptake (pattern I), and a poorly circumscribed, heterogeneous area of uptake (pattern II).^12^ Although the majority of tumors with homogeneous uptake patterns had well‐circumscribed margins in our study, this was not applicable to all tumors. Moreover, poorly circumscribed margins were only seen in about half of the tumors with heterogenous uptake. Based on this, the previously reported classification scheme^12^ might not be suitable to describe scintigraphic findings in the majority of thyroid tumors. We suggest describing thyroid scintigraphic findings by characterization of uptake, uptake pattern, and tumor circumscription separately, rather than constraining to fixed combinations of aforementioned features.

In human medicine, thyroid diseases can produce abnormal patterns that could be described as homogeneous or heterogeneous, increased or decreased, and diffuse or local.^13^ Areas of focally increased uptake (hot nodules) are almost always benign (hyperfunctioning autonomous adenomas), whereas areas of focally decreased uptake (cold nodules) could reflect malignancy.^13^, ^22^ This is in contrast to our findings in dogs, in which most thyroid carcinomas showed (diffusely or focally) increased radionuclide uptake.

In our study, there was a significant association between uptake pattern and hormone status, with the majority of euthyroid dogs having a heterogeneous uptake at the level of the thyroid tumor. It might be hypothesized that loss of cellular pertechnetate trapping is a result from loss of thyroid cell differentiation,^23^ and that a heterogeneous uptake pattern is caused by the presence of both differentiated and dedifferentiated tumor tissue, or both dedifferentiated tumor tissue and remaining normal thyroid tissue. Euthyroid hormone status could be maintained in both scenarios. On the contrary, to acquire a hyperthyroid hormone status, an adequate amount of well‐differentiated tissue, capable of thyroid hormone production, is required. This could explain the larger amount of homogeneous uptake patterns in hyperthyroid dogs, which is also seen in other studies.^12^ If the thyroid tumor destroys enough of the thyroid gland to cause hypothyroidism, the remaining normal tissue will have increased uptake because of increased TSH secretion, which could explain the heterogeneous uptake patterns in hypothyroid dogs.^4^ On the other hand, a heterogeneous uptake pattern could be seen if the dog was previously hypothyroid and the resulting chronic exposure to TSH would cause excess promotion of residual follicular tissue and formation of thyroid follicular neoplasia.^24^ In addition, extensive necrosis and hemorrhage is present in most thyroid tumors, and these areas are expected to have decreased radionuclide uptake, possibly contributing to a heterogeneous uptake pattern.

Importantly, most of the scintiscans were performed using ^99m^TcO_4_. In 6 dogs, both ^99m^TcO_4_ and ^123^I planar scintigrams were performed. In contrast to ^99m^TcO_4_, ^123^I is also organified by the thyroid, allowing for determination of “true” uptake, which could be more reflective of thyroid physiology.^13^, ^14^ Although no differences were seen in uptake or uptake pattern between the ^99m^TcO_4_ and ^123^I scintigrams, it would be interesting to evaluate ^123^I scintigram findings in a larger number of dogs.

A significant association between tumor circumscription and hormone status was found in our study, with well‐delineated tumors in the majority of hyperthyroid and hypothyroid dogs. In a previous study, 5 out of 6 hyperthyroid dogs showed well‐circumscribed uptake,^12^ whereas only 1 out of 3 hypothyroid dogs had a well‐delineated tumor. Another study describes irregular margins in 6 out of 7 masses, but thyroid hormone status is not mentioned in our study.^8^ A more recent study suggests different scintigraphic appearances based on tumor type, with a diffuse increased uptake in follicular tumors or adenomas, a decreased uptake, effacing the normal thyroid tissue, in stromal tumors, and irregular, heterogeneous uptake in mixed cell tumors.^5^ However, in both our study and a previous study,^12^ there was no association between uptake pattern and histologic type of the tumor.

In our study, 3 of 5 dogs with MTCs showed increased radionuclide uptake, which was an unexpected finding because normal and malignant parafollicular C‐cells do not have the ability to trap iodine. In 1 dog that was previously described by our research group,^25^ ^123^I uptake was present in both the primary tumor and thoracic metastases. The remaining 4 dogs had ^99m^TcO_4_ SPECT imaging performed, which was suggestive of vascular activity in 2 dogs and negative for thoracic metastases in 2 dogs. To the authors' knowledge, radionuclide uptake by a medullary carcinoma has only been described in 1 previous report in veterinary medicine.^12^ In human medicine, trapping of radioiodine and ^99m^TcO_4_ has been described,^26^, ^27^ although the mechanism underlying the ability of medullary carcinoma cells remains unclear. Because MTCs may be difficult to distinguish from compact FTCs by histopathology, IHC for calcitonin or markers of neuroendocrine tissue (chromogranin A) is required for their identification.^2^, ^28^ On the other hand, thyroglobulin can be used as a marker for FTCs. In 1 of the MTCs that showed increased pertechnetate uptake in our study, IHC was not available, making the diagnosis of MTC questionable. In the other MCT with increased pertechnetate uptake, IHC was positive for calcitonin, but thyroglobulin staining was not performed. The presence of a mixed medullary‐follicular thyroid carcinoma, which has been described as a rare thyroid tumor in human medicine,^29^, ^30^ can therefore not be excluded. The same applies to the MTC that showed ^123^I uptake; which was positive for chromogranin A, but had no staining for thyroglobulin performed. Further studies, using comprehensive histopathology and IHC to correctly characterize thyroid tumor type, are needed to evaluate possible radionuclide uptake by MTCs.

According to a previous study, planar scintigraphy does not appear to offer any additional benefit, compared to thoracic radiography, for detection of pulmonary metastases.^12^ In our study, only 2 of 29 dogs had radiographic evidence of pulmonary metastases, which is considerably lower than reported metastatic rates.^1^, ^2^, ^3^ One dog, of which the primary tumor had minimal radionuclide uptake, had radiographic but not scintigraphic evidence of pulmonary metastases. It was suggested that the lack of trapping function was similar for both the primary tumor and the metastatic lesion.^12^ In our study, 1 dog had radiographic evidence of metastatic disease whereas thoracic SPECT imaging was negative. The primary tumor of this dog showed a decreased uptake. However, of the 15 dogs that did show metastases on SPECT imaging, 7 had an uptake at the level of the primary mass that was decreased or comparable to the salivary glands, an observation that has also been described for planar imaging.^8^ Therefore, SPECT imaging is valuable for metastasis detection regardless of the uptake of the primary tumor. A possible explanation for the discrepancy between the uptake at the level of the primary mass and metastases could be that the function of the sodium iodide symporter, which is responsible for radionuclide uptake, disappears at the level of the tumor during the course of the disease (loss of differentiation), whereas it could be preserved at the level of metastases, that may display a different pattern of expression of thyroid‐specific proteins.^22^, ^31^

Our results showed that SPECT imaging is a viable imaging technique to screen for thoracic metastasis. Compared to thoracic radiography, SPECT imaging appeared to be especially valuable in detection of thoracic midline lesions. Single‐photon emission computed tomography identified metastatic lesions in 15 dogs, whereas radiographs identified metastatic disease in only 3 of these dogs. In addition, in most cases of thyroid tumors, negative SPECT results seemed to be reliable to exclude metastatic disease. Thoracic CT images were only available for 12 dogs, limiting the possibility to compare SPECT and CT findings. Although the pattern and localization of abnormal thoracic foci of radioiodine uptake were unlikely to be the result of physiologic uptake, false‐positives could not be completely excluded. The use of hybrid SPECT/CT imaging would improve the accuracy of disease detection, because the fusion of the SPECT imaging information with the anatomic CT data provides better identification and interpretation of the foci of pertechnetate/radioiodine uptake, more correct anatomic localization and characterization, and thus more precise differentiation between physiologic uptake and metastatic lesions.^16^, ^17^, ^18^, ^20^, ^32^ Foci corresponding to malignant lesions seen on SPECT/CT in human patients with differentiated thyroid carcinoma are not always identified on diagnostic CT, emphasizing the limitations of CT for confirming the presence of metastatic disease.^17^ Although SPECT is widely used in human medicine, the limited availability in veterinary medicine is a drawback of this technique.

The main limitation of our study is its lack of consistent imaging. Thoracic CT images were only available in part of the dogs, precluding the confirmation or exclusion of the presence of metastatic disease as diagnosed by SPECT imaging. To definitively confirm the presence of metastases, necropsy would have been required, which was not performed in the majority of dogs.

## CONFLICT OF INTEREST DECLARATION

Authors declare no conflict of interest.

## OFF‐LABEL ANTIMICROBIAL DECLARATION

Authors declare no off‐label use of antimicrobials.

## INSTITUTIONAL ANIMAL CARE AND USE COMMITTEE (IACUC) OR OTHER APPROVAL DECLARATION

Authors declare no IACUC or other approval was needed.

## HUMAN ETHICS APPROVAL DECLARATION

Authors declare human ethics approval was not needed for this study.

## Supporting information


**Figure S1** ^99^mTcO_4_ SPECT imaging showing the presence of multiple pulmonary metastases and 1 focus in the thoracic midline

**Figure S2** ^99^mTcO_4_ SPECT imaging showing the presence of a metastasis in the thoracic midline

**Figure S3** ^99^mTcO_4_ SPECT imaging showing the presence of 2 pulmonary metastases
