# Supplementary figures and images for: Planar and single‐photon emission computed tomography imaging in dogs with thyroid tumors: 68 cases
Source: J Vet Intern Med. 2020 Sep 26;34(6):2651–9. doi: 10.1111/jvim.15908 (PMC7694792; doi:10.1111/jvim.15908)

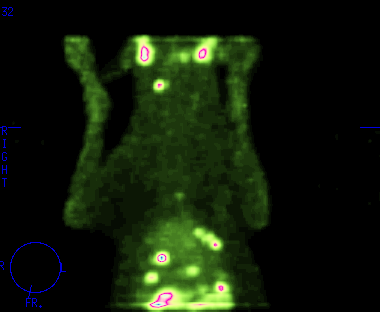

Supplement: Supplementary file 1 — Figure S1 99mTcO4 SPECT imaging showing the presence of multiple pulmonary metastases and 1 focus in the thoracic midline [file JVIM-34-2651-s001.gif]

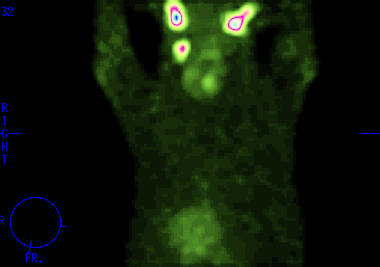

Supplement: Supplementary file 2 — Figure S2 99mTcO4 SPECT imaging showing the presence of a metastasis in the thoracic midline [file JVIM-34-2651-s002.gif]

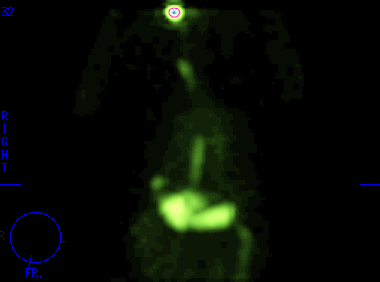

Supplement: Supplementary file 3 — Figure S3 99mTcO4 SPECT imaging showing the presence of 2 pulmonary metastases [file JVIM-34-2651-s003.gif]
